# Drug Delivery System for Emodin Based on Mesoporous Silica SBA-15

**DOI:** 10.3390/nano8050322

**Published:** 2018-05-12

**Authors:** Tamara Krajnović, Danijela Maksimović-Ivanić, Sanja Mijatović, Dijana Drača, Katharina Wolf, David Edeler, Ludger A. Wessjohann, Goran N. Kaluđerović

**Affiliations:** 1 Institute for Biological Research “Siniša Stanković”, University of Belgrade; Bulevar Despota Stefana 142, 11060 Belgrade, Serbia; tamara.krajnovic@ibiss.bg.ac.rs (T.K.); nelamax@ibiss.bg.ac.rs (D.M.-I.); sanjamama@ibiss.bg.ac.rs (S.M.); dijana.draca@ibiss.bg.ac.rs (D.D.); 2 Department of Bioorganic Chemistry, Leibniz Institute of Plant Biochemistry, Weinberg 3, D-06120 Halle (Saale), Germany; katharina.wolf@ipb-halle.de (K.W.); david.edeler@ipb-halle.de (D.E.); ludger.wessjohann@ipb-halle.de (L.A.W.)

**Keywords:** emodin, SBA-15, apoptosis, autophagy, melanoma

## Abstract

In this study mesoporous silica SBA-15 was evaluated as a vehicle for the transport of cytotoxic natural product emodin (EO). SBA-15 was loaded with different quantities of EO (SBA-15|EO1–SBA-15|EO5: 8–36%) and characterized by traditional methods. Several parameters (stabilities) and the in vitro behavior on tumor cell lines (melanoma A375, B16 and B16F10) were investigated. SBA-15 suppresses EO release in extremely acidic milieu, pointing out that EO will not be discharged in the stomach. Furthermore, SBA-15 protects EO from photodecomposition. In vitro studies showed a dose dependent decrease of cellular viability which is directly correlated with an increasing amount of EO in SBA-15 for up to 27% of EO, while a constant activity for 32% and 36% of EO in SBA-15 was observed. Additionally, SBA-15 loaded with EO (SBA-15|EO3) does not disturb viability of peritoneal macrophages. SBA-15|EO3 causes inhibition of tumor cell proliferation and triggers apoptosis, connected with caspase activation, upregulation of Bax, as well as Bcl-2 and Bim downregulation along with amplification of poly-(ADP-ribose)-polymerase (PARP) cleavage fragment. Thus, the mesoporous SBA-15 is a promising carrier of the water-insoluble drug emodin.

## 1. Introduction

Quinones represent a large family of compounds having diverse biological properties. Emodin (EO; 1,3,8-trihydroxy-6-methylanthraquinone) belongs to a group of molecules with an anthraquinone core, isolated from the roots and barks of many plants, molds, and lichens [1,2]. Its multiple biological activities can be ascribed to its affinity to modulate different enzymes, but also to intercalate in DNA leading to the inhibition of the action of topoisomerase II [3]. Reports about its genotoxicity/mutagenicity are still controversial [2,4,5]. On the other hand, numerous studies proved its medicinal value such as laxative, antimicrobial, immunosuppressive, hepatoprotective, cardiotonic, vasorelaxant, and antitumor properties [6]. A wide spectrum of its different effects is related to similar signaling pathways involved in the regulation of essential physiological processes in mammalian cells. For example, inhibition of PTK signaling pathways by EO will reflect on the cellular response to different growth factors, cytokines, or hormones [7]. Its antiinflammatory effect is assigned to inhibition of inducible nitric oxide synthase (iNOS), cytokine production, prostaglandin synthesis, and super oxide production [6]. By influencing the redox status, EO indirectly modulates signal transduction [2,6]. However, EO behaves as a scavenger and inducer of oxidative stress depending on the circumstances. Therefore, EO will interfere with proliferation, differentiation, motility or dying signals in cells. Its efficacy in vitro and in vivo in numerous types of cancer is well documented [2,6,8,9,10,11,12]. Multiple mechanisms have been described as possible modes of the antitumor action of EO. Alkylation of DNA or cellular constituents leads to perturbation of cell cycle. Emodin inhibited HER-2/neu tyrosine kinase activity, suppressed growth and induced differentiation of HER-2/neu-overexpressing breast cancer cells in vitro and in vivo [13]. It is well described that EO is able to induce apoptotic cell death in numerous cancer cell lines: tongue squamous, cervical, pancreatic, breast, colon, leukemia, lung, and hepatocellular cells [2,6,8,9,10,11,12]. Induced apoptosis is primarily exhibited through the mitochondrial pathway, as shown by the activation of caspases-3, -9, and cleavage of poly-(ADP-ribose)-polymerase (PARP). Apart from the influence on the viability of tumor cells, EO could affect migratory and invading properties of different tumor cells through inhibition of MMP-9, MMP-2, focal adhesion kinase (FAK), ERK1/2, and Akt/PKB activation, and partial inhibition of AP-1 and NF-kB transcriptional activities [6,14,15,16,17,18]. The fact that EO is a substrate for multidrug resistant pumps enables it to compete with applied chemotherapeutics enhancing their intracellular accumulation [19]. Therefore, EO is able to sensitize tumor cells to some chemotherapeutics (e.g., cisplatinol (abiplastin), doxorubicin (adriablastin), 5-FU, arsenic trioxide, gemcitabine) [2,20,21].

Despite a broad range of biological effects, the physicochemical properties of EO represent a boundary for its application. Its low bioavailability is caused by low water solubility, low oxidative stability as well as intensive biotransformation [22,23]. Moreover, long-term use of EO can lead to its accumulation and generate many undesirable side effects like renal tubule adenoma, renal failure and liver cancer [24]. The mentioned limits represent a challenge for scientists to improve its delivery and effectiveness using different approaches such as loading to polylactic acid microspheres, lipid nanoparticles or modifying them by glycosylation [22,23,24]. One of the possibilities for improving delivery and targeting is the utilization of mesoporous silica nanoparticles (SBA-15). SBA-15 mesoporous silica has already been shown as a suitable drug delivery system for various potent antitumor compounds [25,26,27]. Vavsari et al. summarized research related to the SBA-15 as drug delivery system used not only for anticancer agents (i.e., doxorubicin, gemcitabine, desatinib), but also for bisphosphonate (zoledronic acid, sodium alendronate), antidiabetic (metformin), antifungal drugs (itraconozole), antibiotics (i.e., cephalexin, gentamicin, sulfadiazine), as well as vehicle for various proteins, enzymes and hormones [28]. Superiority of SBA-15 as drug delivery system for titanium(IV) complexes in comparison to MCM-41 was observed [29]. Recently a great potential of SBA-15 particles in delivery could be pointed out for some metal-based drugs. Namely, SBA-15 loaded with organotin(IV) compound completely abolished tumor growth in vivo, while compound alone was not found to be efficient under same experimental settings [30]. SBA-15 carrying cisplatin is able to induce phenotype changes in B16F10 melanoma cells transforming them in nonmalignant phenotype [31]. Also, preparation of SBA-15 loaded with low amount of EO demonstrated promising properties of this mesoporous silica as carrier [32].

The release of the drug from SBA-15 is generally regulated by diffusion. Thus, the release profile may be tuned, for example, by modulating pore diameter [33]. Moreover, MSNs with 2D hexagonally ordered pore channels hinder diffusion of drug. On the other hand, the interaction of the drug with the silica pore wall should not be underestimated [34]. Hence, the interaction between functionalities present in the pore wall as well as in drug itself may play important role in drug release. The release of hydrophobic drug (i.e., itraconazole) from the hydrophilic SBA-15 material is guided, depending on the low or high loaded drug amount, by displacement desorption (drug ↔ water) or by dissolution of crystalline or amorphous drug from the pores, respectively [35].

The aim of this study is to achieve loading of high EO amount in SBA-15 and evaluate antitumor potential of SBA-15|EO particles in three different melanoma cell lines: less malignant mouse B16, metastatic clone of B16 (B16F10) and highly aggressive human A375. This approach should offer not just better solubility, and stability, but also efficient cellular uptake of EO, bypassing the cellular efflux of the compound [36]. On the other hand, the increased “permeability and retention effect” enables mesoporous silica nanoparticles (MSN) to selectively enter the tumor tissue and to retain within the cells. Their size prevents their extravasation from blood vessels and thus accumulation in normal tissues [37].

## 2. Results and Discussion

### 2.1. Preparation and Characterization of SBA-15 Containing Emodin 

SBA-15 is prepared by a standard sol-gel procedure and afterwards calcined [38]. Then, SBA-15 was activated (vacuum, 150 °C, 16 h) and used for loading of different quantities of EO. Initially, loading of EO (ratio: SBA-15, 100 mg; EO, 10 mg) was performed at room temperature (→ SBA-15|EO1) and at 60 °C (→ SBA-15|EO1a) to check the temperature dependency on the loading efficiency. In both cases used conditions showed a high and comparable efficiency of drug loading in SBA-15 (at room temperature: 88.2%, at 60 °C: 90.4%). As similar loading was found for both MSNs all other materials were prepared at room temperature. The ratio of SBA-15 and EO was varied (SBA-15, always 100 mg; EO, 20, 30, 40, 50 or 60 mg; SBA-15|EO1 → SBA-15|EO5, respectively) and materials with different loadings were gained. The content of EO in SBA-15 for these different materials was 8.1–36.4% with efficiency of 88.2–95.6% (HPLC, see experimental part), contrarily to previously described SBA-15|EO where only 0.7% of EO was loaded into MSNs.

The yellow powdery products of the SBA-15|EO*n* (*n* = 1, 1a and 5) were characterized with energy dispersive X-ray spectroscopy (EDX), SEM, TEM nitrogen adsorption-desorption isotherms, small-angle X-ray scattering experiments (SAXS) and IR spectroscopy.

**Figure 1:**
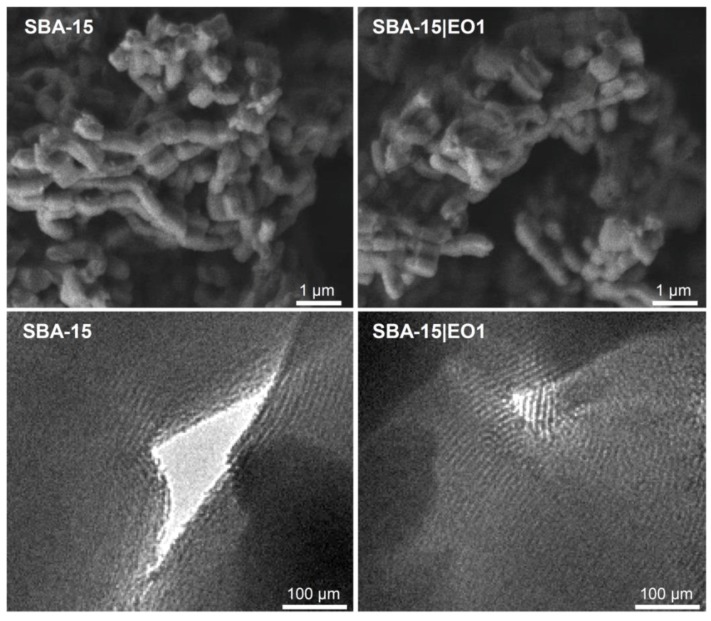
SEM (**upper**) and TEM images (**lower**) of SBA-15 and SBA-15|EO1, as example.

Morphological properties of MSNs were determined using SEM and TEM imaging. The cylindrical shape (ca. 650 ± 70 × 440 ± 50 nm) of materials could be clearly seen with the help of SEM (Figure 1). A 2D hexagonal structure with an order at the mesoscopic scale could be identified by TEM measurements (Figure 1) [39]. The tubes of the mesoporous material are almost continuous for the full length of the particles. Moreover, these fragile tube systems were not damaged under the loading conditions. SEM and TEM images did not show changes upon loading of SBA-15.

**Figure 2:**
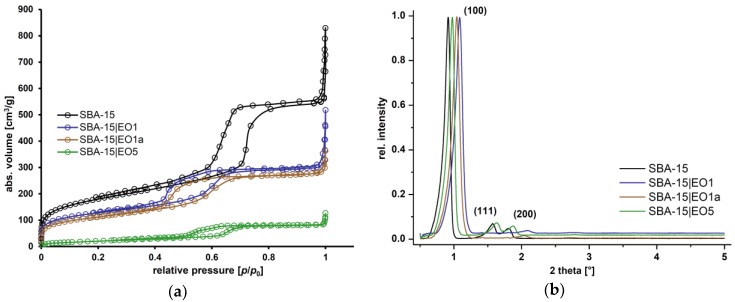
N_2_ adsorption-desorption isotherms (**a**) and small-angle X-ray scattering (SAXS) patterns (**b**) of SBA-15 and SBA-15|EO*n* (*n* = 1, 1a and 5).

The nitrogen sorption isotherms and SAXS experiments are given in Figure 2. SBA-15 exhibits characteristic type IV isotherm behavior and the hysteresis loop shows type H1 behavior (IUPAC classification) [40] and a narrow size distribution, which indicates a typical mesostructure with open channels. However, upon loading of EO into SBA-15 isotherms and hysteresis loop changed. As expected, the specific surface area (*S*_BET_), determined from nitrogen sorption measurements, was decreased upon loading. SBA-15 loaded with EO showed a decrease of the surface area which is higher in ratio in the case of SBA-15|EO5, as is expected due to the higher amount of EO in its structure (*S*_BET_ = 651 m^2^·g^−1^, SBA-15; *S*_BET_ = 79 m^2^·g^−1^, SBA-15|EO5). The mean pore diameter, calculated using the BJH method [41], as well as the pore volume were reduced upon loading. Accordingly, the wall thickness of the MSNs increased. These data evidence that EO has been effectively adsorbed into the SBA-15 interior channels.

SAXS analysis was used for the characterization of MSNs and the obtained patterns are shown in Figure 2b. For all materials, the expected and well-defined structure of SBA-15 was observed and corresponds to a mesoscopic order assigned to mesoporous materials. The SAXS patterns show three peaks, which can be indexed on the 2D hexagonal lattice with the *d-*spacing values between 43 and 50 Å. MSNs displayed a well resolved pattern, with the highest intensity of diffraction peak at ca. 1.0° corresponding to the (100) plane. Considerable reduction in the intensity of the diffraction peaks was detected upon loading of EO due to a blocking of the micropores and shrinkage in inner pore dimensions by the organic molecules [42].

IR spectra show typical bands for asymmetric (1061 cm^−1^) [43] and symmetric stretching vibrations of Si–O–Si (ring structures; 806 cm^−1^), as well as symmetric bending vibrations (443 cm^−1^). Nanomaterials containing EO exhibit additional vibrations belonging to C=O (625 cm^−1^) from the active component EO.

### 2.2. Stability and Drug Release Studies

Administration of therapeutic drugs is preferably oral because this circumvents discomfort and danger of infection compared to parenteral administration. This enlarges patient compliance [44]. Regularly, drug degradation takes place by hydrolysis, thus acidic conditions (e.g., gastric juice) might have a great impact on drug decay. The stability of EO in acidic media has already been evaluated in the literature [45].

**Figure 3:**
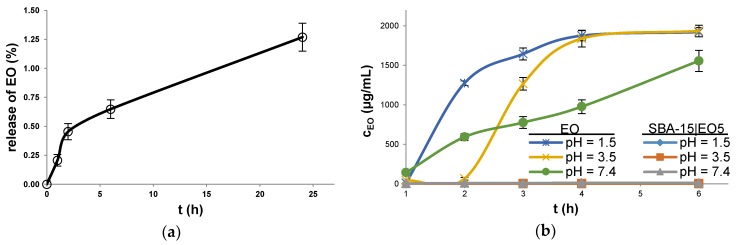
(**a**) Release profile of EO from SBA-15|EO5 in PBS during 24 h; (**b**) Solubility of EO, alone and from SBA-15|EO5, under pH 1.5, 3.5 and 7.4.

Soaking of SBA-15|EO5 at the physiological pH (7.4) for different times (0, 1, 2, 6 and 24 h) and subsequent quantification of EO revealed that drug release occurs very slowly and in a small amount (Figure 3a). Thus within 24 h only 1.3% of EO is released from SBA-15. Contrary, previously described SBA-15 loaded with low amount of EO (ca. 0.7%) releases ca. 90% of the drug under same pH and upon 24 h [46]. In SBA-15 containing ca. 0.7% EO, drug is dispersed as monolayer on the walls of SBA-15 and fast release of drug takes place [46]. Besides, the release profile correlated to the pore diameter and volume. The particles of SBA-15|EO5 having high EO content (36.4%) and those reported previously with low EO content (ca. 0.7%) have 4.36 and 6.45 nm pore diameter and 0.13 and 0.92 cm^3^ g^−1^ pore volume, respectively. Smaller pore diameter and low pore volume of SBA-15|EO5 point out the fact that EO is deposited in mesoporous channels contrary to previously reported EO loaded in SBA-15. Based on results reported herein and literature data, it could be assumed that EO in SBA-15|EO5 is rather deposited as crystalline or amorphous form inside nanopore channels from where a slow release follows [35,47].

In order to assess the effect of acidic conditions present in stomach (pH = 1.5–3.5) on the EO stability, the behavior of the active component as well as SBA-15|EO5 in simulated gastric juice pH as well as under physiological pH (pH = 1.5, 3.5 and 7.4) was tested and analyzed by HPLC (Figure 3b). Soaked solution of EO at pH 1.5 showed the presence of the drug already after 1 h of shaking, along with several degradation products in small quantity (Figure S1). The amount of solubilized EO increased almost 140 times after 2 h. At pH 3.5 the amount of dissolved EO raised during 2 h by the factor of ca. 1.5. From 3 up to 6 h at pH 1.5 and 3.5 solubilized EO reached concentration of almost 2 mg/mL. The difference in solubilized EO and its solubility (2.7 mg/mL) could be explained by the fact that EO is decomposing at mentioned acidic conditions, which is consistent with the HPLC findings where degradation products were observed. Investigations at physiological pH (7.4) showed that EO is dissolving and reaching ca. 1.5 µg/mL of EO after 6 h. Comparing to experiments at pH 1.5 and 3.5 fewer degradation products could be detected. In contrast, the amounts of released EO from SBA-15|EO5 soaked for 6 h at pH 1.5 and 3.5 were found to be in traces (below the quantification limit of HPLC). Thus, almost all EO loaded remained in the pores of SBA-15, circumventing interaction with acidic media and its degradation. Moreover, EO from SBA-15|EO5 is released very slowly over 6 h at pH 7.4, and fewer degradation products could be detected, in direct comparison to EO used as such. Due to the fact that EO in SBA-15|EO5 is deposited as crystalline or amorphous form inside nanopore channels, the release of EO occurs very slowly.

Furthermore, it is well known that EO is photolabile when exposed to visible light [48]. In order to investigate if degradation occurs, both EO and SBA-15|EO5 were exposed to light for 2 and 24 h (see Figure S2). The probes with EO showed more degradation products than that of SBA-15|EO5, proving that SBA-15 to some extent protects EO from degradation. Herein, it is proved that EO loaded in SBA-15 is at lower extent degraded than when EO alone exposed to light or to various tested pH as well as forced degradation as described in the literature [45].

### 2.3. In Vitro Studies

**Figure 4:**
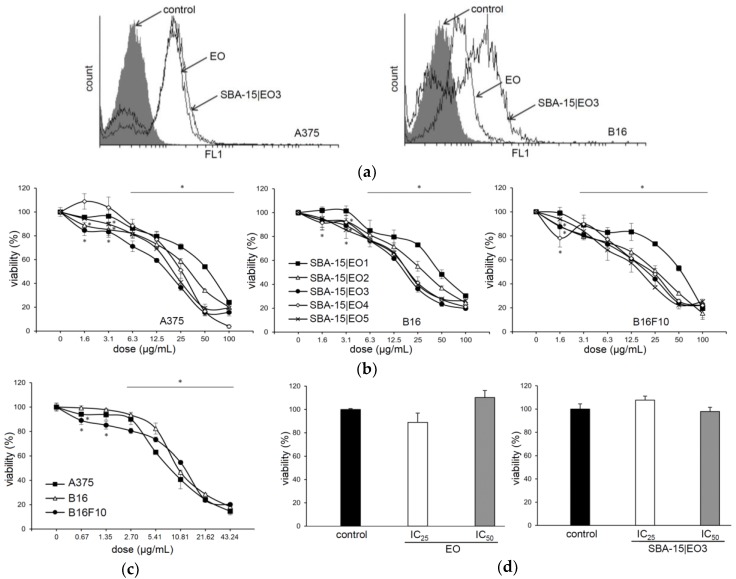
Free emodin (EO) and EO loaded into SBA-15 decrease the viability of melanoma cell lines without disturbing viability of peritoneal macrophages. (**a**) A375 and B16 EO and SBA-15|EO3 uptake (48 h). (**b**) Cell viability in the presence of SBA-15|EO (MTT assay, 48 h). (**c**) Cell viability in the presence of EO (MTT assay, 48 h). (**d**) The viability of macrophages in the presence of EO and SBA-15|EO3 (CV assay, 48 h).

To evaluate the efficacy of emodin loaded into SBA-15, human melanoma A375, mouse melanoma B16 and its metastatic subclone B16F10 were exposed to a wide range of doses of original drug and six different modalities of SBA-15 with raising concentrations of EO for 48 h (Figure 4). For comparison, SBA-15 alone was tested against the same tumor cell lines (see Figure S3). Materials with the lowest EO content prepared at different temperatures (SBA-15|EO1 and SBA-15|EO1a, r.t. and 60 °C, respectively) showed similar MC_50_ values against tested cell lines (results not shown). Dose dependent decrease of cellular viability correlated with increasing amount of EO within SBA-15 carrier with a saturation at ca. 27% (Table 1). Loading of the drug into nanomaterial conserved and even potentiated its activity. EO is a naturally occurring compound whose cellular uptake is easy to follow due to its autofluorescence feature. Therefore, the decrease of cellular viability correlated with the efficient internalization of EO from free as well as loaded form (Figure 4a–c). While tumor cells were highly sensitive to the treatments, the viability of mouse macrophages was not disturbed upon exposure to both EO and SBA-15|EO3 in IC_50_ concentrations determined on tumor cells (Figure 4d). It is obvious that the novel nanomaterial shows some selectivity toward malignant cells. The potential of EO to disturb the viability of different tumor cells is well described [2,6,8,9,10,11,12,47]. However, it is known that MSNs are taken by the cells through endocytosis, so it is clear that macrophages as professional phagocytes uptake MSNs efficiently [49]. Since intracellular targets of EO loaded into SBA-15 are tightly connected with cell ability to proliferate, the viability of nonstimulated primary macrophages, as low proliferative cells, was not diminished.

**Figure 5:**
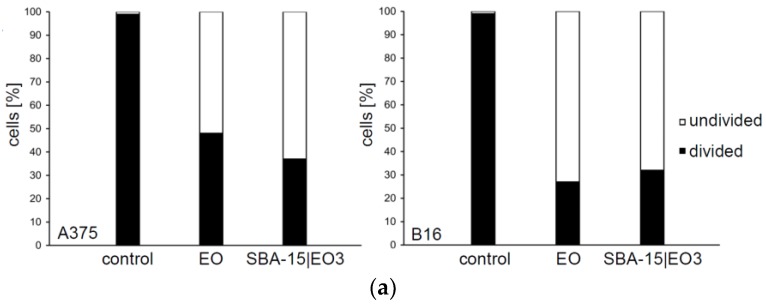

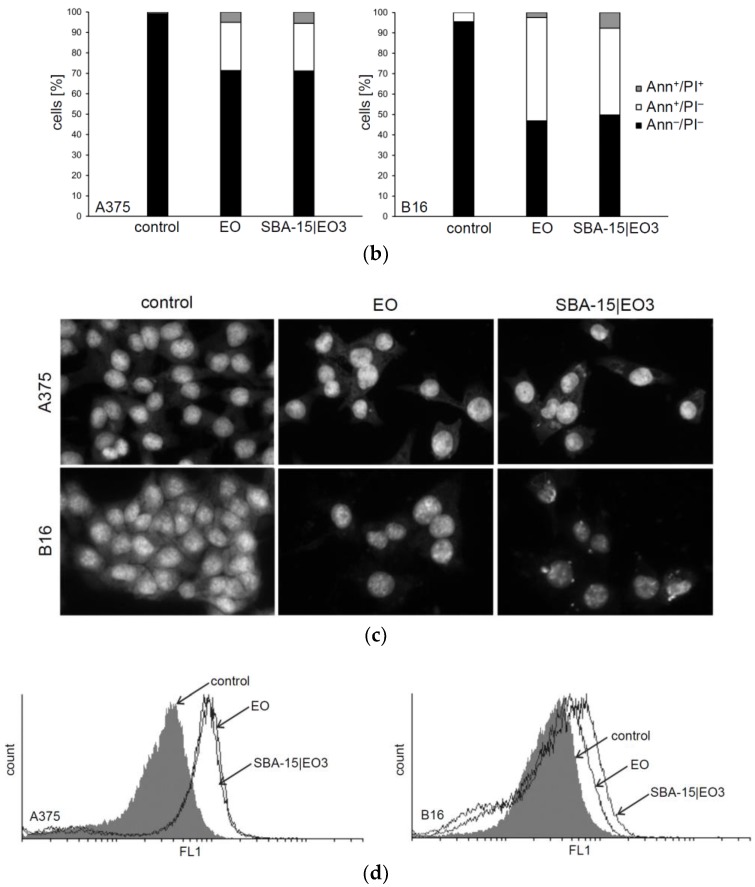
EO and SBA-15|EO3 induce caspase dependent apoptosis. (**a**) Cellular proliferation; (**b**) Ann/PI double staining; (**c**) Assessment of apoptotic cell morphology; (**d**) Caspase activity.

Measurement of cellular proliferation revealed that treatment of EO and SBA-15|EO3 influences the division of cells (Figure 5a). The percentage of undivided subpopulations was remarkably higher upon both treatments in comparison to control on A375 and B16 cell lines. As a consequence of inhibited proliferation, triggered apoptotic cell death was detected. After 48 h long treatment with IC_50_/MC_50_ doses of both EO and SBA-15|EO3, an elevated percentage of early (Ann^+^PI^–^) and late apoptotic (Ann^+^PI^+^) cells was detected (Figure 5b). Concordantly, apoptotic cell death promoted by the agents was visualized by PI staining revealing typical morphological signs of this process (Figure 5c). Decreased nuclear volume, unregular shape of nuclei as well as condensed chromatine are visible upon the treatment with both EO and SBA-15|EO3. However, these features were more obvious upon the treatment with EO loaded MSN. In parallel, analysis of cell cycle distribution confirmed the presence of hypodiploid cells with fragmented DNA upon the 48 h treatment of A375 and B16 cells with both EO and SBA-15|EO3 (Figure S4). In compliance with the data from microscopical evaluation of PI stained cells, the effect was stronger in cultures exposed to SBA-15|EO3. Total caspase activity was then analyzed by flow cytometry of Apostat stained cells. Simultaneously, enhanced caspase activity was found (Figure 5d) in both cell types after the treatment with EO in either, free or nanoform. Obtained results clearly indicated that apoptotic process triggered by experimental therapeutics, independently from MSN packaging, is classical caspase dependent form. This finding was in concordance with previously described potential of EO to activate caspase dependent apoptosis [2,6,8,9,10,11,12].

Having in mind the quantity of EO in SBA-15|EO3 (B16: MC_50_ = 19.7 µg/mL → IC_50_ = 20.0 µM) and active concentration of EO alone (B16: IC_50_ = 42.0 µM), it is clear that loading amplified its activity (for IC_50_/MC_50_ values see Table 1). Since the apoptosis is often followed by autophagy, especially in autophagy prone cells such as A375, the presence of autophagosomes in the cytoplasm of cells exposed to both compounds, using acridin orange staining, was evaluated. Treatment with SBA-15|EO3 elevated the percentage of cells with acidic vesicles (8.2%) in A375 but not in B16 cells. Since co-treatment with specific inhibitors of this process, 3-methyl adenine and chloroquine, and SBA-15|EO3 did not result in viability recovery (see Figure S5), it is clear that in this setting autophagy was not cytoprotective but rather slightly contributed to drug mediated toxicity. Induction of autophagy upon the exposure to a similar anthroquinone, aloe emodin was described previously in glioma cells with the same tendency [50]. Obviously, nanopackaging of emodin did not change the mechanism of its action.

**Figure 6:**
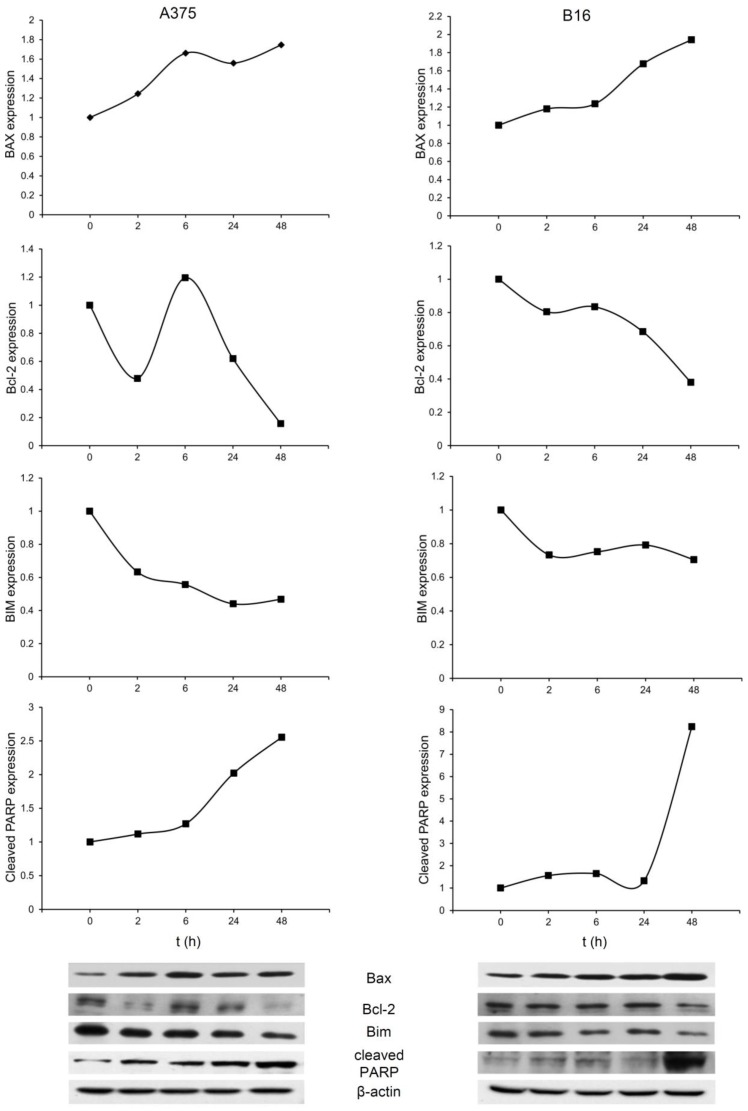
SBA-15|EO3 upregulated pro- and down-regulated antiapoptotic mediators. Protein expression of Bax, Bcl-2, Bim and poly-(ADP-ribose)-polymerase (PARP) fragment were analyzed in different time points by western blot.

As mentioned above apoptosis as the mechanism of EO action is observed in pancreatic, breast, colon, leukemia, lung, hepatocellular carcinoma, tongue squamous, and cervical carcinoma [2,6,8,9,10,11,12,47]. Therefore, the molecular profile of apoptotic process in response to SBA-15 loaded with EO was evaluated (Figure 6). The intracellular response of both tested cell lines, independent from differences in their origin and invasiveness, was quite similar in terms of expression of main proapototic and antiapoptotic molecules. As expected, upregulated expression of proapoptotic Bax synchronized with diminished expression of antiapoptotic Bcl-2 in both cell lines was detected. The capability of EO to interfere with the Bax/Bcl-2 ratio is already described in breast and colon cell lines [51,52]. Reduced Bim expression is one of the general features of melanoma cells, and at least partly responsible for the establishment of an apoptosis resistant phenotype [53,54,55].

Upon the exposure of tested cell lines to emodin incorporated in mesoporous silica, Bim additionally decreased but this effect did not compromise the completion of the apoptotic process triggered by the compound. Having in mind that Bax is efficiently upregulated even if Bim is missing, implicated Bim as an independent alternative loop involved in Bax release and transport to the mitochondrial membrane. Together with this, decreased Bcl-2 expression in cells of both cell lines allowed propagation of the apoptotic signal. Finally, amplified presence of cleaved PARP fragment confirmed that apoptosis is realized through a defined route as described in literature [56].

## 3. Materials and Methods

### 3.1. Materials and Methods

Pluronic 123 (P123) and TEOS (Sigma, Taufkirchen, Germany), and ЕО (TCI chemicals, Eschborn, Germany) were purchased and used as received from the distributor.

### 3.2. Characterization of the Mesoporous Silica Nanoparticles

SEM and TEM imaging were performed on JEM-1010 (JEOL). EDX experiments were conducted on electron microscopes JSM-6701F and JSM-7500F (JEOL) as well as XL 30 ESEM-FEG (Philips, Eindhoven , The Netherlands). NOVA 1000 (Quantachrome, Odelzhausen, Germany,) was employed for nitrogen adsorption-desorption measurements. IR spectra were recorded on a Nicolet 5700 FT-IR (Thermo, Madison, WI, USA) spectrometer in the range 4000–400 cm^−1^ (KBr). X-ray measurements (SAXS) were performed on a D8 ADVANCE (Bruker, Karlsruhe, Germany) X-ray diffraction system.

### 3.3. Preparation of SBA-15

Pluronic P123 (16.0 g) was dissolved in water (120 mL) and HCl (2 M, 320 g). Afterwards TEOS (35.2 g) was added dropwise. The reaction mixture was stirred for 20 and 24 h at room temperature and 80 °C, respectively [32]. The obtained material was filtered and wasted with water (3 × 100 mL). Subsequently the material was heated (1 K min^−1^) to 500 °C and calcified for 24 h. Yield: 9.0 g; BET surface: 651.03 m^2^ g^−1^; pore volume: 0.87 cm^3^ g^−1^; pore diameter: 5.41 nm; wall thickness: 4.32 nm; IR: *ν* = 1085 (br, vs; Si–O–Si), 807 (w; Si–O–Si), 463 cm^−1^ (vs; Si–O–Si); XRD (2θ in °, Miller indices): 0.9197 (100), 1.5703 (111), 1.8116 (200); lattice parameter (nm): 9.7; crystal size (nm): 84.

### 3.4. Preparation of SBA-15 Loaded with Emodin (EO)

EO was suspended in toluene (20 mL). To the activated SBA-15 (dried under vacuum for 6 h at 160 °C) a suspension of EO was added and the mixture was stirred at room temperature (or in case of SBA-15|EO1a at 60 °C) for 48 h. For the used amount of EO and SBA-15 see details below in appropriate SBA-15|EO*n*. Afterwards, the suspension was filtered and the isolated material was washed successively with toluene (2 × 10 mL). The obtained materials were dried under vacuum at room temperature and lyophilized for 24 h. Filtrate was evaporated and used for HPLC quantification of non-loaded EO (the difference between the amount of EO used for preparation of nanomaterials and non-loaded amount of EO present the quantity of loaded EO).

SBA-15|EO1: EO 30 mg, 300 mg SBA-15; Yield: 300.22 mg; Loading (HPLC): 8.10%; Efficiency: 88.2%; BET surface: 458.34 m^2^·g^−1^; pore volume: 0.48 cm^3^·g^−1^; pore diameter: 3.55 nm; wall thickness: 5.03 nm; IR: *ν* = 1634 (C=O), 1086 (br, vs; Si–O–Si), 802 (w; Si–O–Si), 462 cm^−1^ (vs; Si–O–Si); XRD (2θ in °, Miller indices): 1.0456 (100), 2.0320 (200); lattice parameter (nm): 8.6; crystal size (nm): 66.

SBA-15|EO1a: EO 30 mg, 300 mg SBA-15; Yield: 244.00 mg; Loading (HPLC): 8.29%; Efficiency: 90.4%; BET surface: 402.87 m^2^·g^−1^; pore volume: 0.43 cm^3^·g^−1^; pore diameter: 3.56 nm; wall thickness: 4.71 nm; IR: *ν* = 1628 (C=O), 1084 (br, vs; Si–O–Si), 804 (w; Si–O–Si), 465 cm^−1^ (vs; Si–O–Si); XRD (2θ in °, Miller indices): 1.0876 (100), 2.0844 (200); lattice parameter (nm): 8.3; crystal size (nm): 64.

SBA-15|EO2: EO 15 mg, SBA-15 50 mg; Yield: 56.70 mg; Loading (HPLC): 21.86%; Efficiency: 93.3%.

SBA-15|EO3: EO 20 mg, SBA-15 50 mg; Yield: 51.42 mg; Loading (HPLC): 27.41%; Efficiency: 94.4%.

SBA-15|EO4: EO 25 mg, SBA-15 50 mg; Yield: 53.53 mg; Loading (HPLC): 32.20%; Efficiency: 95.0%.

SBA-15|EO5: EO 180 mg, SBA-15 300 mg; Yield: 372.48 mg; Loading (HPLC): 36.45%; Efficiency: 95.6%; BET surface: 78.79 m^2^·g^−1^; pore volume: 0.13 cm^3^·g^−1^; pore diameter: 4.36 nm; wall thickness: 5.04 nm; IR: *ν* = 1627 (C=O), 1088 (br, vs; Si–O–Si), 803 (w; Si–O–Si), 465 cm^−1^ (vs; Si–O–Si); XRD (2*θ* in °, Miller indices): 0.9722 (100), 1.6332 (111), 1.8641 (200); lattice parameter (nm): 9.3; crystal size (nm): 76.

### 3.5. Stability and Drug Release Studies

Release of EO from SBA-15|EO5 was assessed suspending SBA-15|EO5 (5 × 5 mg) in solution (1.0 mL) at physiological pH (7.4). Obtained samples were shaken for 0, 1, 2, 6 and 24 h at 25 °C. Afterwards, supernatant was separated by centrifugation and amount of EO was quantified using HPLC as described in Section 3.6.

A stock solution of EO (2 mg) in DMSO (1 mL) was prepared. 100 µL stock was transferred to solutions conditioned at different pH (1.5, 3.5 and 7.4; 900 µL). SBA-15|EO5 (15 × 5 mg) was suspended in solutions (1.0 mL) with different pH (1.5, 3.5 and 7.4). Working solutions (EO) and suspensions (SBA-15|EO5) were shaken for 1, 2, 3, 4 and 6 h at 25 °C. The probes were centrifuged (SBA-15|EO5) and content of EO in supernatant was analyzed by HPLC as described in Section 3.6.

Solid EO (2 mg) and SBA-15|EO5 (5 mg) were illuminated with 150 W halogen lamp (Heimwerker Mobillicht, Schabe) at the distance 0.3 m for 2 and 24 h. The probes were treated with acetonitrile (1 mL), centrifuged (only SBA-15|EO5) and analyzed by HPLC as described in Section 3.6.

### 3.6. HPLC Determination of EO

EO from the filtrates, obtained from the SBA-15 loading procedures (non-loaded EO; see Section 3.4), or released quantity of EO from the supernatant (see Section 3.5) were determined by HPLC (Agilent 1260 Infinity bestehend) equipped with auto sampler (G1329), column heater (G1316A), quaternary pump (G1315C) and DAD detector (G1315C). For the HPLC experiments, LiChroChart column (125 mm × 4 mm), injection volume 5 µL, mobile phase gradient MeOH/H_2_O (30→100% in 10 min), and detection at λ 254 were used. For calibration five standards prepared in MeOH were used (2.5, 25, 50 and 500 µg/mL).

### 3.7. Reagents and Cells

Fetal calf serum (FCS), RPMI-1640, phosphate-buffered saline (PBS), dimethyl sulfoxide (DMSO), 3-(4,5-dimethylthiazol-2-yl)-2,5-diphenyltetrazolium bromide (MTT), carboxyfluorescein diacetate succinimidyl ester (CFSE), crystal violet (CV), and propidium iodide (PI) were from Sigma (St. Louis, MO, USA). Acridine orange (AO) was obtained from Labo-Moderna (Paris, France). Annexin V-FITC (AnnV) was bought from Santa Cruz Biotechnology (Dallas, TX, USA). Murine melanoma B16, human melanoma A375 and murine metastatic melanoma cells B16F10 were kind gifts from Prof. Siniša Radulović (Institute for Oncology and Radiology of Serbia), Prof. Ferdinando Nicoletti (Department of Biomedical Sciences, University of Catania, Catania, Italy) and Prof. Barbara Seliger (Institute for Medical Immunology, Martin Luther University Halle-Wittenberg), respectively. Cells were regularly cultivated in HEPES-buffered RPMI-1640 medium supplemented with 10% FCS, 2 mM L-glutamine, 0.01% sodium pyruvate and antibiotics (culture medium) at 37 °C in a humidified atmosphere with 5% CO_2_. Cells were seeded at 5 × 10^3^ cells/well in 96-well plates for viability determination and 2.5 × 10^5^ cells/well in 6-well plates for flow cytometry and Western blot analysis. Peritoneal resident macrophages were obtained from C57BL/6 mice by peritoneal lavage with ice cold PBS. Cells were counted and seeded at 2 × 10^5^/well in 96-well plates and incubated overnight. Before treatment, nonadherent cells were removed.

### 3.8. Preparation of Drug Solutions

EO was dissolved in DMSO at 100 mM and kept at −20 °C until use. Various SBA-15|EO (2 mg/mL) were suspended in culture medium directly before use and working solutions were made in culture medium immediately before treatment.

### 3.9. MTT and CV Test

Cells were cultivated in the presence of different concentrations of EO, SBA-15 alone and various SBA-15|EO for 48 h. For the CV test, cells were fixed with 4% paraformaldehyde for 10 min at RT and subsequently stained for 15 min with 1% CV solution. Then cells were washed with tap water, dried and the dye was dissolved in 33% acetic acid. For the MTT test, viable cells were incubated in MTT staining solution (0.5 mg/mL) for approximately 1 h, the dye was removed and produced formazan was dissolved in DMSO. The absorbance was measured with an automated microplate reader at 540 nm with a reference wavelength of 670 nm. The results are expressed as a percentage of control values obtained in untreated cultures [57].

### 3.10. Uptake of EO

Cells were exposed to IC_50_/MC_50_ dose of EO or SBA-15|EO3, respectively, for 48 h, detached and analyzed with CyFlow^®^ Space Partec using the Partec FloMax^®^ software (Partec GmbH, Münster, Germany).

### 3.11. Annexin V-FITC/PI, Acridin Orange and Apostat Staining

Cells were incubated with IC_50_/MC_50_ dose of EO or SBA-15|EO3 for 48 h. Then cells were detached, counted and split for three assays. Cells were stained with AnnV-FITC/PI or Apostat according to the manufacturer’s protocol. For AO staining cells were incubated for 15 min at 37 °C in 1 µg/mL of dye solution, washed and resuspended in PBS. Cells were analyzed with a CyFlow^®^ Space Partec using PartecFloMax^®^ software.

### 3.12. Carboxyfluorescein Succinimidyl Ester (CFSE) Staining

For detection of cellular proliferation, cells were stained with 1 µM of carboxyfluorescein succinimidyl ester (CFSE) for 10 min at 37 °C, and then exposed to IC_50_/MC_50_ dose of EO or SBA-15|EO3 for 48 h. At the end of cultivation, cells were washed, trypsinized, dissolved in PBS and analyzed as mentioned above.

### 3.13. Morphological Assessment of Apoptosis

For morphological assessment of apoptotic cells, cells were seeded at 2 × 10^4^/well on a chamber slide overnight, and incubated for 48 h in the absence or presence of the drugs. After 15 min of fixation with 4% paraformaldehyde at room temperature, cells were stained with 20 μg/mL PI in 0.1% Triton X-100, 0.1 mM EDTA pH 8.0, and 50 μg/mL RNase in PBS, washed in PBS and prepared for fluorescence microscopy by covering with 50% glycerol in PBS.

### 3.14. Cell Cycle Analysis

B16 and A375 cells were seeded in 6-well plates (2.5 × 10^5^/well) and incubated with the IC_50_/MC_50_ dose of EO or SBA-15|EO3, respectively for 48 h. After the incubation period, cells were fixed in 70% ethanol and stored at 4 °C overnight. The next day cells were washed twice with PBS and then stained with PI (20 μg/mL) in the presence of RNase (0.1 mg/mL) for 45 min at 37 °C in the dark. The distribution of cells among different cell cycle phases was analyzed by FACSCalibur flow cytometer using Cell Quest Pro software.

### 3.15. Western Blot Analysis

A375 and B16 cells were cultivated with an IC_50_/MC_50_ dose of EO and SBA-15|EO3 for 2, 6, 24 and 48 h and then lysed in protein lysis buffer (62.5 mM Tris-HCl (pH 6.8 at 25 °C), 2% (*w/v*) SDS, 10% glycerol, 50 mM dithiothreitol). Proteins were further electrophoretically separated on 10–12% SDS-polyacrylamide gels. Electrotransfer to polyvinylidenedifluoride membranes at 5 mA/cm^2^ was done with a semidry blotting system (Fastblot B43; BioRad, Göttingen, Germany). Membranes were blocked with 5% (*w/v*) BSA in PBS and subsequently incubated overnight at 4 °C in the presence of specific antibodies to Bax (E63), Bim (Y36), beta-actin (all from Abcam, Cambridge, UK), PARP, Bcl-2 (Cell Signaling Technology, Danvers, MA, USA). As a secondary antibody, goat anti-rabbit IgG-HRP (Santa Cruz Biotechnology, Dallas, TX, USA) was used. For detection of bands, the chemiluminescence detection system (ECL; GE Healthcare, Chalfont St. Giles, Buckinghamshire, UK) was used.

### 3.16. Statistical Analysis

The results of cell viability estimation calculated from triplicate observations are presented as mean ± SD from one representative of at least three experiments. Analysis of variance (ANOVA) together with a Student-Newman-Keuls test were used for assessment of significance of the differences between treatments and p values of less than 0.05 were considered as significant.

## 4. Conclusions

Herein preparation and characterization of mesoporous silica SBA-15 loaded with various quantities of EO (→ SBA-15|EO1–SBA-15|EO5: 8–36%) is presented. SBA-15 protects EO even in extremely acidic media (pH = 1.5–3.5) and from photodecomposition (24 h). In vitro investigations on human melanoma A375, as well as mouse melanoma B16 and B16F10 cells showed a dose dependent decrease of cell viability which is also in direct relationship with the EO content in SBA-15. SBA-15|EO3, one of the most active mesoporous silica particles loaded with EO, does not influence peritoneal macrophage viability. On the other hand, SBA-15|EO3 hinders tumor cell proliferation. Additionally, the material triggers caspase dependent apoptosis synchronized with overexpression of Bax as well as down-regulation of Bcl-2 and Bim. Consequently, cleaved PARP fragment is amplified. All of this demonstrates positive effects of SBA-15 as drug carrier system. SBA-15 amplifies the activity of EO in vitro and protects the active compound from spontaneous, light promoted inactivation or degradation driven by extremely acidic conditions corresponding to gastric pH.

## Figures and Tables

**Table 1:** IC_50_ (µM) and MC_50_ (µg/mL) values of free EO and loaded into SBA-15 material after 48 h of treatment.

Cell Line	Assay	IC_50_ (µM)	MC_50_ (µg/mL)				
		EO	SBA-15|EO1	SBA-15|EO2	SBA-15|EO3	SBA-15|EO4	SBA-15|EO5
A375	MTT	32.30 ± 1.30	57.95 ± 1.77	38.45 ± 6.43	21.07 ± 5.58	24.47 ± 1.08	22.30 ± 1.84
	CV	39.70 ± 3.24	60.80 ± 3.11	42.47 ± 1.99	24.33 ± 0.55	27.67 ± 6.57	23.97 ± 2.56
B16	MTT	33.45 ± 6.58	49.47 ± 5.23	31.13 ± 4.77	18.23 ± 0.92	20.70 ± 1.64	20.07 ± 1.71
	CV	42.00 ± 3.11	51.13 ± 5.98	35.80 ± 3.03	19.73 ± 2.30	20.83 ± 0.68	13.43 ± 0.06
B16F10	MTT	47.95 ± 2.76	53.40 ± 1.56	26.10 ± 3.12	18.00 ± 3.54	21.63 ± 1.82	19.40 ± 3.39
	CV	54.73 ± 6.40	33.67 ± 0.74	20.60 ± 2.40	17.15 ± 2.19	17.37 ± 5.16	17.30 ± 2.97

EO = emodin.

## Supplementary Materials

The following are available online at http://www.mdpi.com/2079-4991/8/5/322/s1.
