# Supplementary material for: Drug Delivery System for Emodin Based on Mesoporous Silica SBA-15
Source: Nanomaterials (Basel). 2018 May 12;8(5):322. doi: 10.3390/nano8050322 (PMC5977336; doi:10.3390/nano8050322)
Supplement: Supplementary file 1 [file nanomaterials-08-00322-s001.pdf]

nanomaterials

Supplementary Information

Drug Delivery System for Emodin Based on Mesoporous Silica SBA-15

Tamara Krajnović [1], Danijela Maksimović-Ivanić [1], Sanja Mijatović [1], Dijana Drača [1], Katharina Wolf [2], David Edeler [2], Ludger A. Wessjohann [2] and Goran N. Kaluđerović [2,*]

[1] Institute for Biological Research "Siniša Stanković", University of Belgrade; Bulevar Despota Stefana 142, 11060 Belgrade, Serbia; tamara.krajnovic@ibiss.bg.ac.rs (T.K.); nelamax@ibiss.bg.ac.rs (D.M.-I.); sanjamama@ibiss.bg.ac.rs (S.M.); dijana.draca@ibiss.bg.ac.rs (D.D.)

[2] Department of Bioorganic Chemistry, Leibniz Institute of Plant Biochemistry, Weinberg 3, D-06120 Halle (Saale), Germany; katharina.wolf@ipb-halle.de (K.W.); david.edeler@ipb-halle.de (D.E.); ludger.wessjohann@ipb-halle.de (L.A.W.)

* Correspondence: goran.kaluderovic@ipb-halle.de; Tel.: +49-345-5582-1370

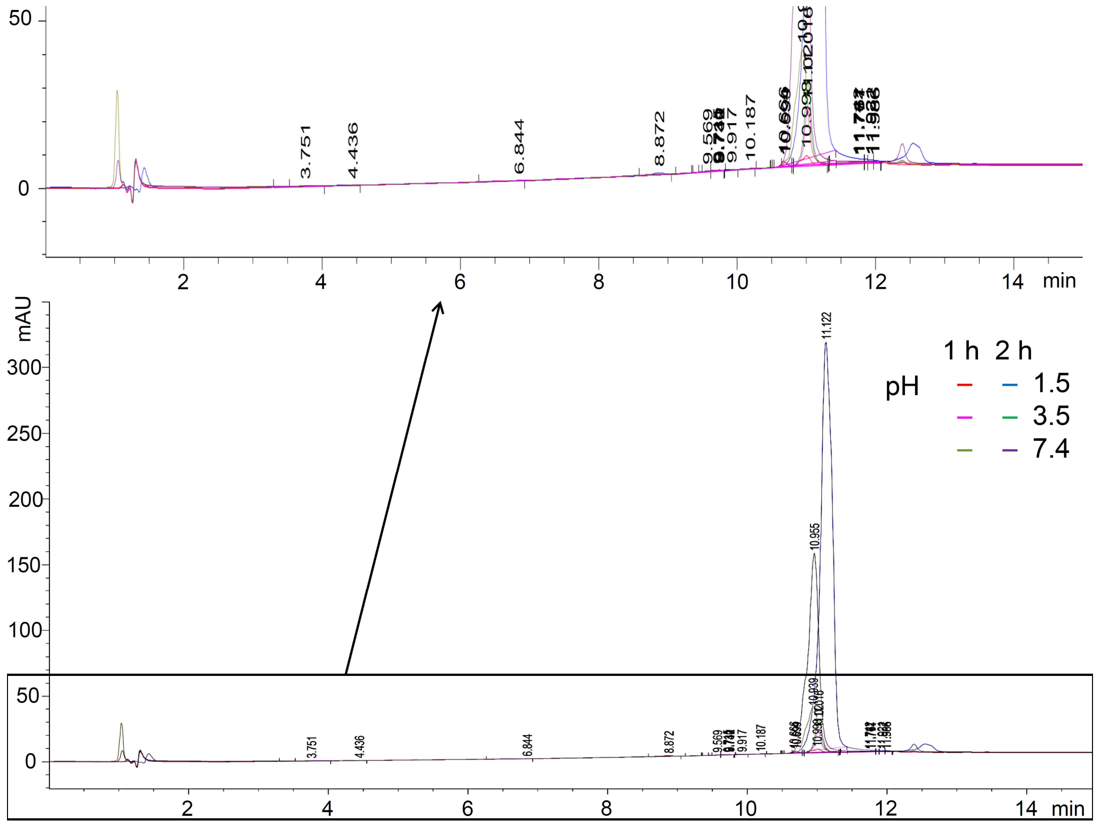

(**a**)

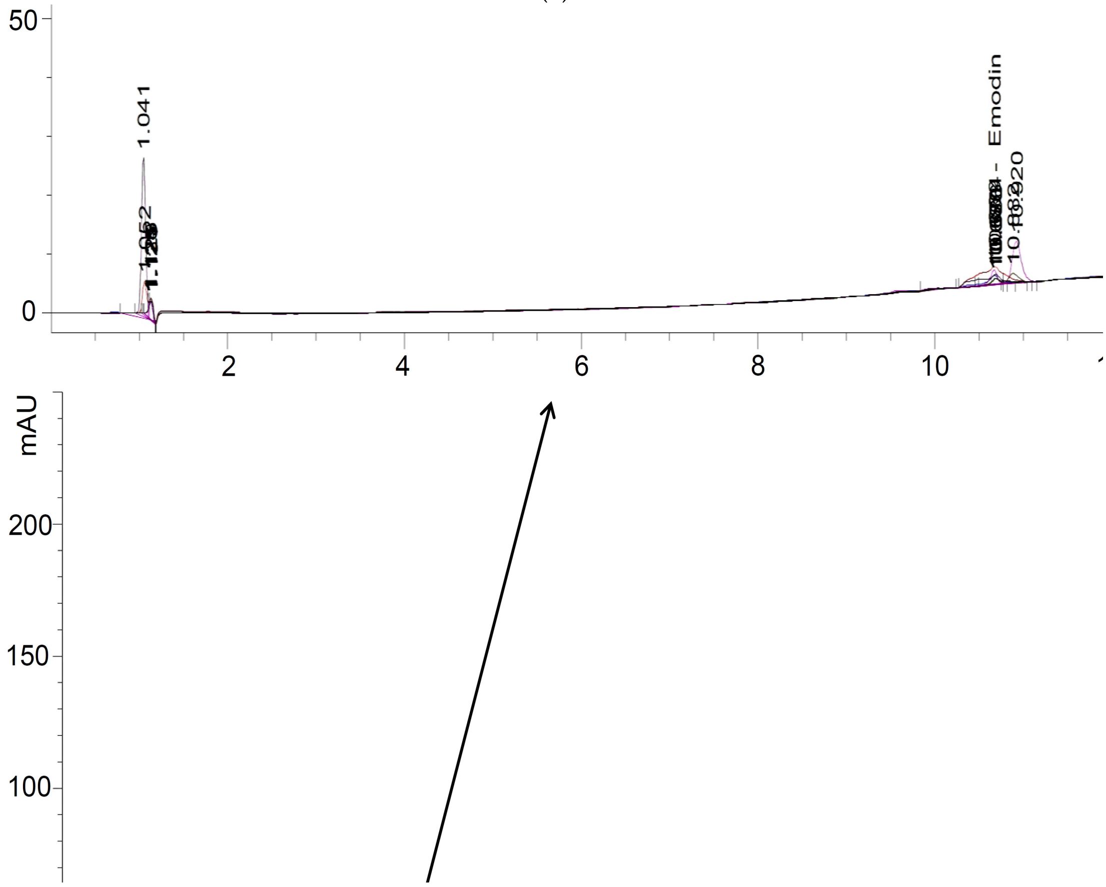

(**b**)

Figure S1. HPL chromatograms of (a) free emodin (EO) or (b) EO loaded into SBA-15 exposed to pH 1.5, 3.5 or 7.4 for 1 or 2 h.

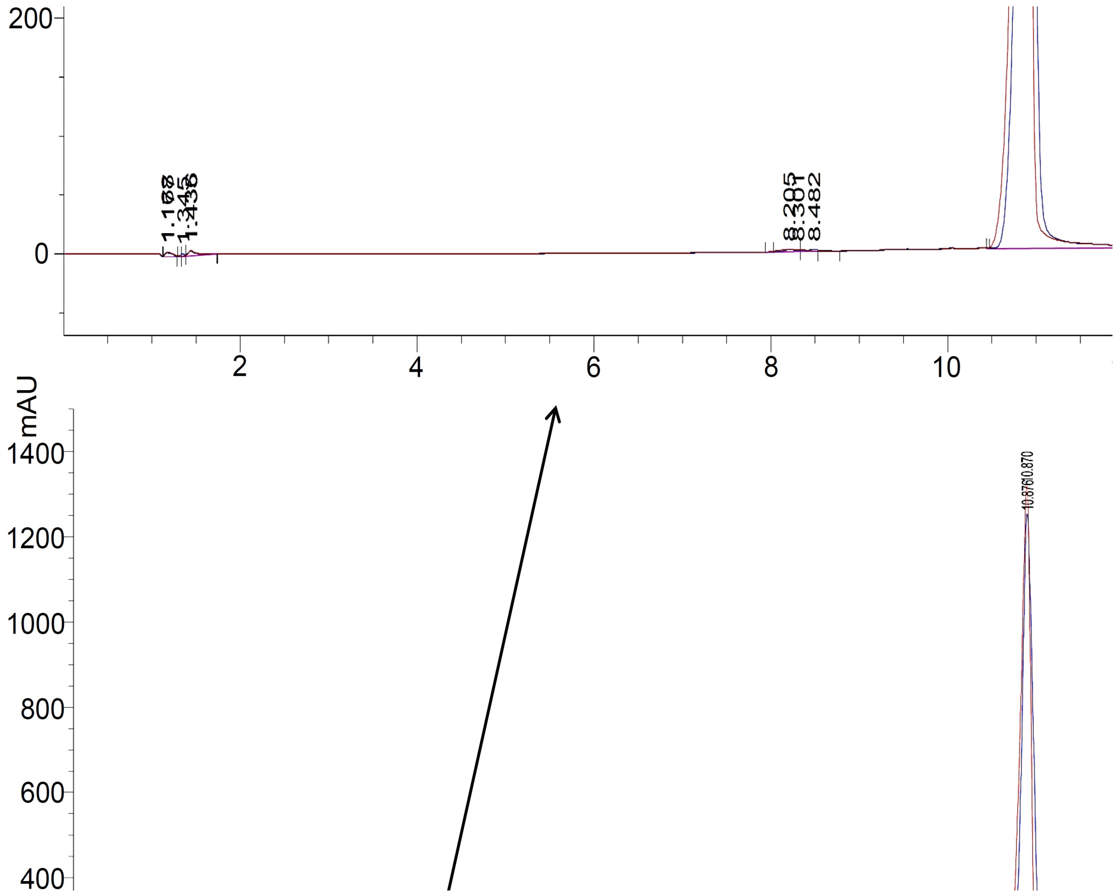

(**a**)

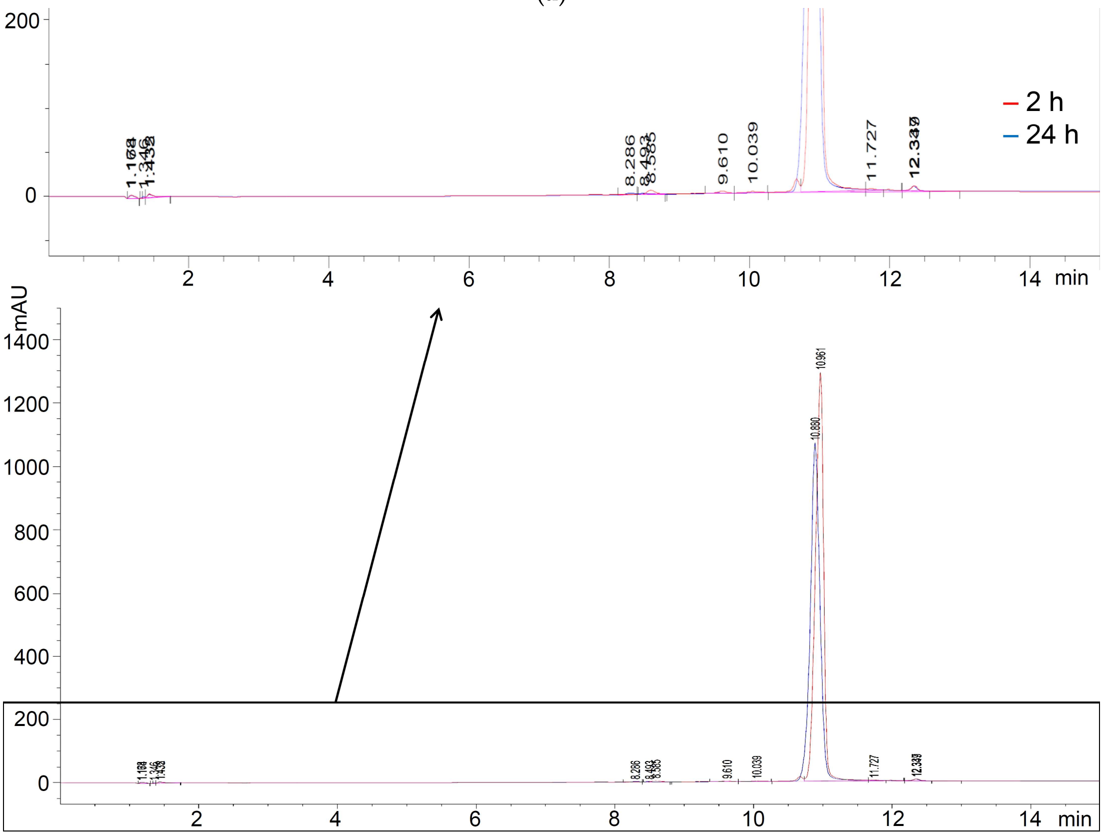

(**b**)

Figure S2. HPL chromatograms of (a) free EO or (b) EO loaded into SBA-15 exposed to light for 2 (red) and 24 h (blue).

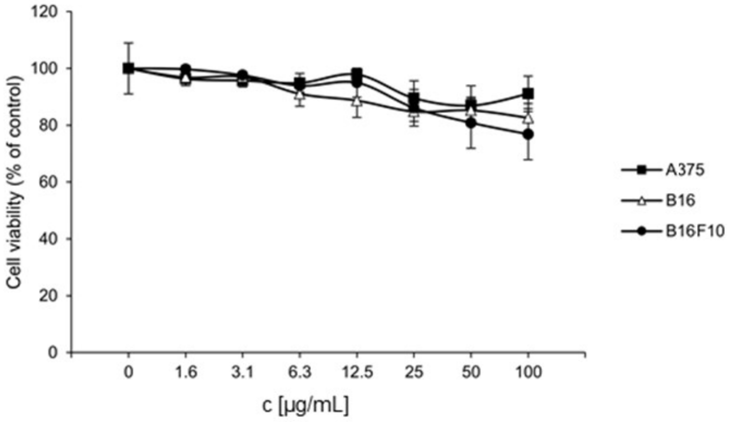

Figure S3. Viability of tumor cells in the presence of various concentrations of SBA-15 (unloaded).

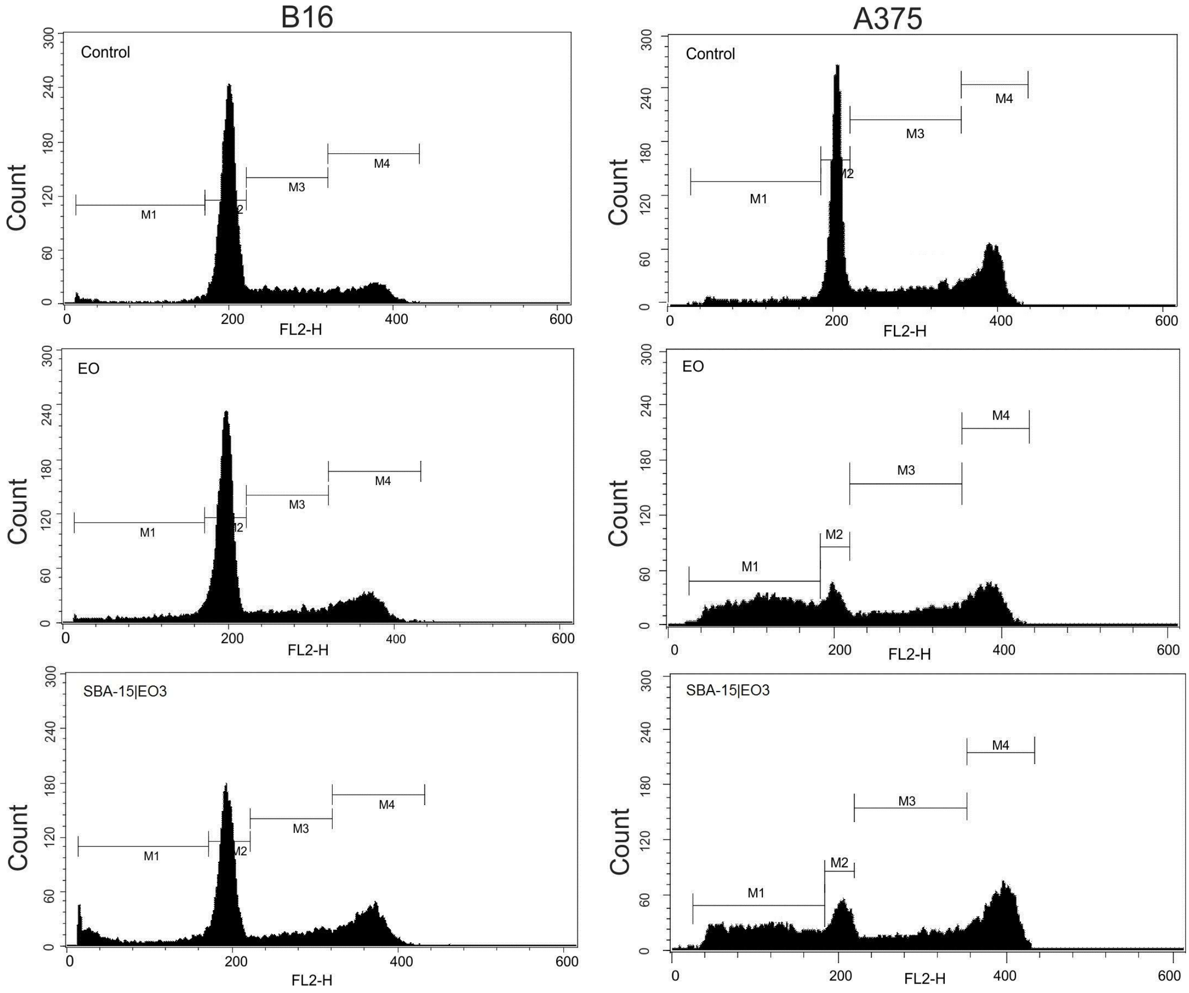

Figure S4. Cell cycle distribution upon the 48 h treatment of B16 and A375 cells with EO or SBA-15|EO3 (M1–M4: sub-G1, G0/G1, S, G2/M, respectively).

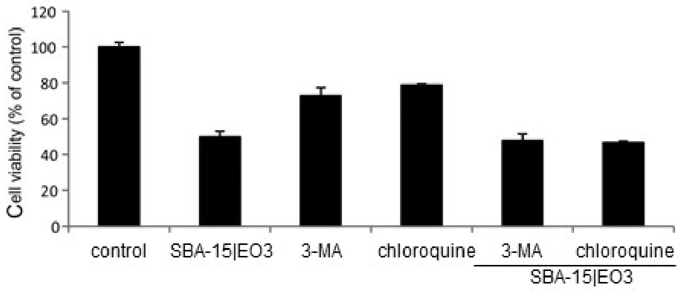

Figure S5. A375 cells treated with SBA-15|EO3 at MC_{50} concentration, in the absence or presence of autophagy inhibitors (3-methyladenine (3-MA) or chloroquine).
